# A Rare Streptomyces griseus Infection of the Sacroiliac Joint: A Case Report

**DOI:** 10.7759/cureus.20078

**Published:** 2021-12-01

**Authors:** Junho Song, Tyler J Humphrey, Andrew Zhang, John K Czerwein, Simon Chao

**Affiliations:** 1 Orthopaedic Surgery, Hospital for Special Surgery, New York, USA; 2 Orthopaedic Surgery, Massachusetts General Hospital, Harvard Medical School, Boston, USA; 3 Orthopaedic Surgery, Louisiana State University Health Sciences Center, Shreveport, USA; 4 Orthoapedic Surgery, Warren Alpert Medical School of Brown University, Providence, USA; 5 Orthopaedic Surgery, Warren Alpert Medical School of Brown University, Providence, USA

**Keywords:** clinical case report, lower back pain (lbp), si joint, septic sacroiliitis, radiculopathy, sacroiliac joint pain, streptomyces griseus, infectious sacroiliitis

## Abstract

A previously healthy 26-year-old female presented with one month of worsening low back pain radiating to the right lower extremity. Magnetic resonance imaging (MRI) without contrast of the lumbar spine demonstrated enhancement of the right sacroiliac joint. Sacroiliac joint aspiration followed by culture and microbiology revealed *Streptomyces griseus* as the cause of infectious sacroiliitis. *Streptomyces griseus* is a part of the normal human flora that produces a plethora of secondary metabolites applied in various medications such as streptomycin. This represents the first described case of infectious sacroiliitis due to *Streptomyces griseus *in the literature. It is critical for spinal surgeons to consider fastidious organisms, such as *Streptomyces griseus*, on the differential diagnosis of sacroiliac joint pain, especially in patients with systemic symptoms and elevated inflammatory laboratory markers.

## Introduction

The sacroiliac (SI) joint is a potential source of lower back pain, contributing to symptoms in 15-30% of such patients [1,2]. SI pain due to infection, however, is exceedingly rare and most often documented in case reports or series. Accurate diagnosis of infectious sacroiliitis can be challenging due to the heterogeneity of the clinical presentation and nonspecific physical examination findings [3]. Physicians often have a low index of suspicion because of the rarity of this pathology, resulting in a delayed diagnosis [4]. Herein, we report the case of radicular pain in a 26-year-old female, ultimately found to be due to *Streptomyces griseus* infection of the SI joint. This represents the first described case of infectious sacroiliitis due to *Streptomyces griseus*.

## Case presentation

A previously healthy 26-year-old female presented to a spine surgery clinic with one month of worsening low back pain and radiating right leg pain. She denied any inciting trauma or drug use. The patient reported sharp, aching, and throbbing pain across her low back, particularly exaggerated over the right SI joint. The severity of the pain was 6/10 at rest and 8/10 with activities. The pain radiated down the right lower extremity, causing occasional numbness and tingling in the toes. The pain was aggravated by both sitting and motion; application of ice alleviated the pain.

The physical examination revealed non-antalgic gait, no tenderness over the spinous processes, and no step-offs or gross deformities. However, there was diminished range of motion of the lumbar spine with tenderness about the paraspinal musculature and point tenderness to the right SI joint. Right-sided straight leg raise and FABER (flexion, abduction, external rotation)/Patrick’s tests were positive. Mild weakness (strength 4/5) was noted in the right tibialis anterior, extensor hallucis longus, and gastrocnemius/soleus. Deep tendon reflexes, sensation, and pulses were normal throughout.

**Figure 1:**
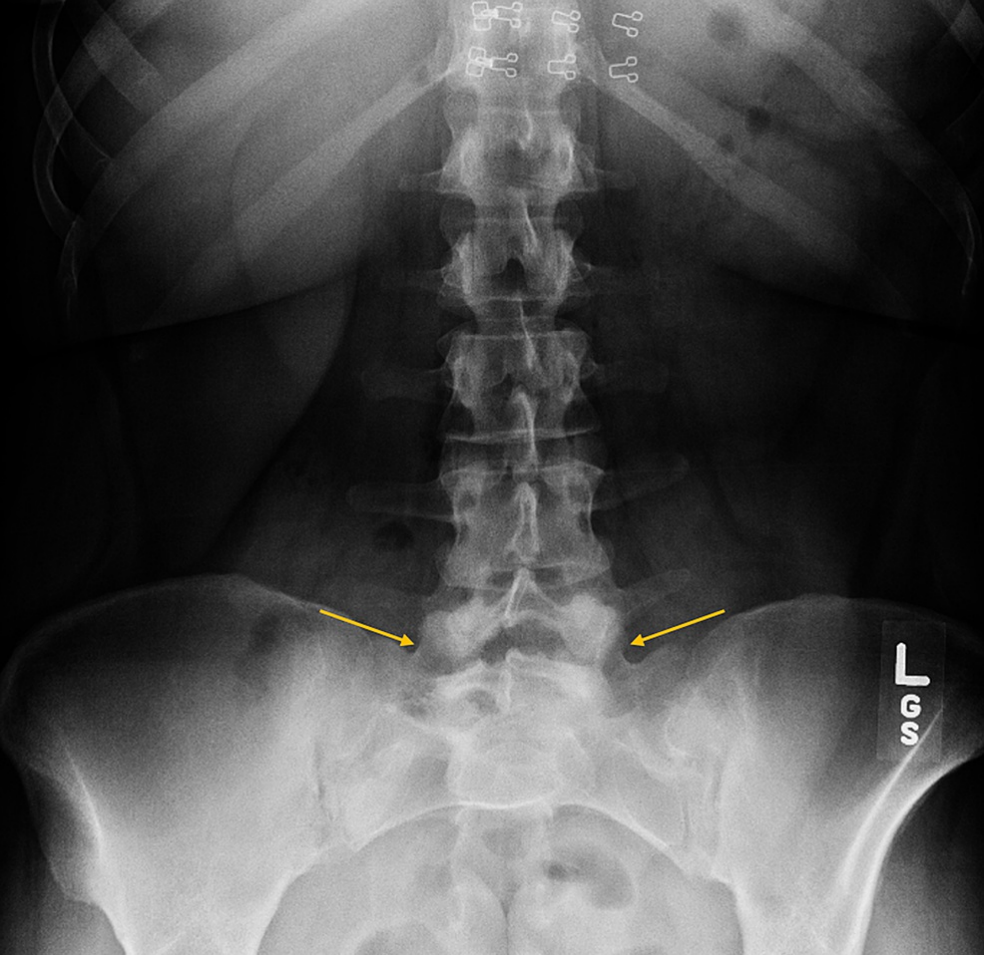
AP X-ray of the lumbar spine and pelvis. AP radiograph revealing transitional lumbosacral anatomy (arrows) and disc degeneration throughout the lumbar segments. AP, anteroposterior.

**Figure 2:**
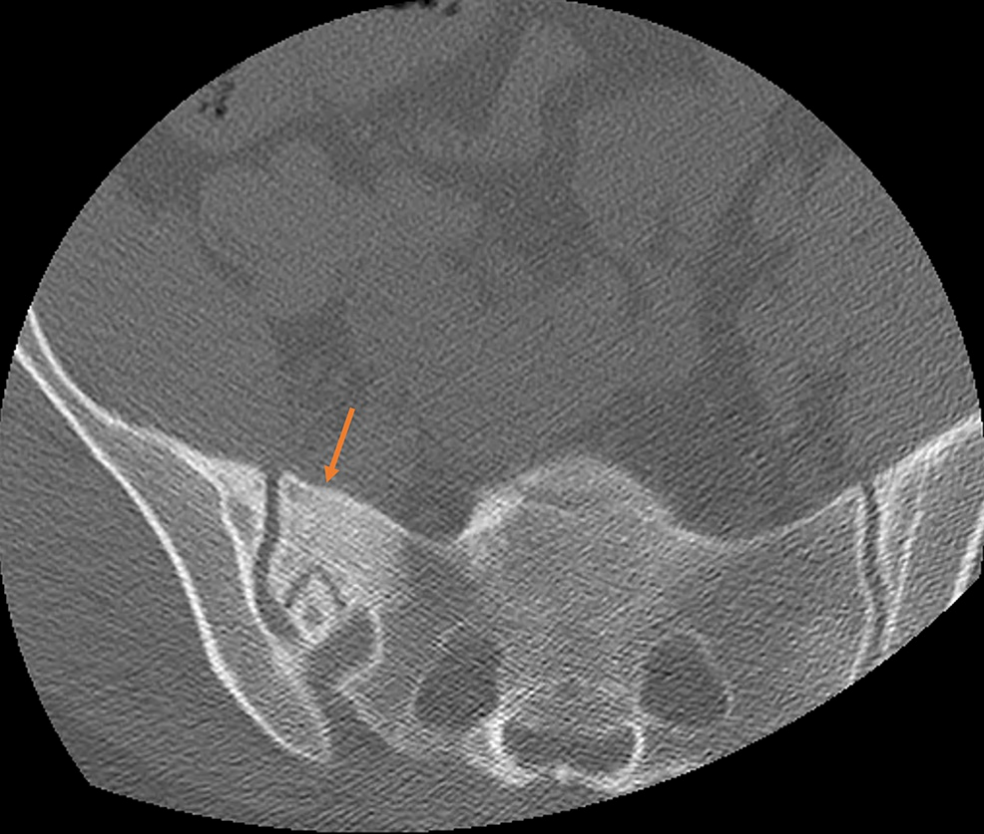
Axial CT scan of the sacrum. Axial CT scan of the sacrum showing hyperdensity of the right sacroiliac region (arrow). CT, computed tomography.

Plain radiographs revealed transitional lumbosacral anatomy, moderate spondylosis, and disc degeneration throughout the lumbar segments, and Grade 1 retrolisthesis at L5-S1 (Figure 1). Computed tomography (CT) scan of the lumbar spine showed sclerosis and lucency of the right SI joint, with suggestion of a possible involucrum (Figure 2).

**Figure 3:**
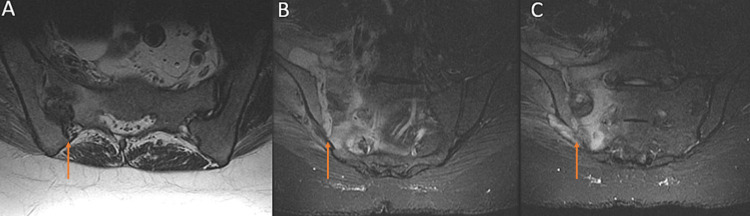
MRI of the sacrum. Axial (A) and coronal (B, C) MRI of the sacrum revealing abnormal right SI joint, with sacral alar marrow signal change and high T2 signal focus (arrows) extending anteriorly to the right iliacus muscle. MRI, magnetic resonance imaging; SI, sacroiliac.

The radicular symptoms prompted further evaluation with a magnetic resonance imaging (MRI) without contrast of the lumbar spine, which was significant for sacral alar marrow signal change and high T2 signal focus extending anteriorly to the right iliacus muscle (Figure 3). This was followed by further laboratory work-up, and a CT-guided biopsy of the right SI joint. Laboratory investigation revealed elevated erythrocyte sedimentation rate 87 mm/h, C-reactive protein 32.7 mg/L, and white blood cell (WBC) count 12.7 cells/mcL. During the biopsy, 7 cc of fluid was aspirated from the joint, which improved the pain. Analysis of the SI joint aspirate revealed an elevated WBC of 247 cells/mm^3^, no crystals, and a preliminary pathology read of a “probable mold.” The aspirate was sent to a quaternary referral center in the US to evaluate for fastidious organisms, ultimately resulting in *Streptomyces griseus* a few months later.

Prior to the final identification of the infectious organism, the patient underwent operative irrigation and debridement at another institution. Unfortunately, the procedure was complicated by methicillin-resistant *Staphylococcus aureus* septicemia, which required prolonged treatment with intravenous (IV) antibiotics. At 2 years of follow-up and without further intervention, the patient reported her symptoms resolved completely with no residual pain or motor deficits.

## Discussion

The SI joint is a large, irregularly shaped diarthrodial joint, stabilized by a thick posterior ligamentous complex. The SI joint is innervated by sensory fibers from a nerve plexus formed by lateral branches of the posterior rami of L5-S4, making it capable of being a significant pain generator [5]. However, the SI joint is often overlooked as a source of back pain due to the multiple other potential sources of pain in the region, such as the lumbar discs, lumbar facets, and piriformis region. Nevertheless, SI joint pain and dysfunction represents a high disease burden and can significantly impact quality of life [6]. SI joint pain can present with symptoms in the lower back, buttocks, pelvis, and sometimes radiating to the ipsilateral lower extremity; thus, its presentation can also mimic radiculopathy [7]. Of note, patients often present with “false-positive” straight leg raise testing, as seen in our patient’s case, while it likely stems from “dural tension” by an inflamed sacrum when the leg is flexed past 60 degrees [8].

Infectious sacroiliitis is rare and often produces misleading clinical signs that delay diagnosis [9,10]. Its incidence is higher in younger individuals, likely due to an age-related reduction in vascularization and mobility of the SI joint, which limits colonization by microorganisms in older patients [11]. It most commonly manifests from hematogenous seeding, so risk factors include infectious endocarditis, immunodeficiency (e.g. congenital disease, iatrogenic immunosuppressive medication, HIV, cancer), and IV drug abuse [8]. Other rare reported etiologies include *Aspergillus fumigatus* and *Mycobacterium tuberculosis* infection; thus, a thorough travel history is warranted in most patients with systemic symptoms (e.g. fever, night sweats, chills) and a physical examination consistent with SI inflammation [12,13].

To our knowledge, we present the first known case in the literature of infectious sacroiliitis due to *Streptomyces griseus*. Most *Streptomyces* infections in humans lead to mycetoma, a chronic suppurative growth on the foot with tumor-like nodule formation and draining sinuses, or, rarely, invasive pulmonary infections in immunocompromised individuals [14]. *Streptomyces griseus* infections are often seen in cats and dolphins with mycetoma [15]. Three prior reports of human *Streptomyces griseus *infection were identified: Chander et al. described a cervicofacial mycetoma, Clarke et al. reported a brain abscess, and Kapadia et al. described a series of five invasive pulmonary infections [15-17]. *Streptomyces griseus* is known to inhabit moist soils, produce a filamentous mycelium, and is the bacterial species from which the antibiotic streptomycin is derived [18]. The filamentous branching pattern of the organism likely explains why the initial pathology report for our patient read “probable mold,” underscoring the importance of sending the aspirate for analysis at highly specialized laboratories for final identification and therefore proper treatment. 

Identifying *Streptomyces griseus* as the infectious source is critical, as its treatment can vary significantly from other aerobic actinomycetes that appear similarly on initial microscopy, such as *Nocardia*. While most *Nocardia* infections are managed with trimethoprim-sulfamethoxazole (TMP-SMX), up to 30% of *Streptomyces griseus* strains are resistant to TMP-SMX and are more susceptible to antibiotics such as doxycycline, minocycline, clarithromycin, and amikacin [18,19]. Thus, empiric management prior to final organism identification should account for both organisms as a possibility. Of note, the CDC reported in a 1990 study that *Streptomyces griseus* accounted for almost 8% of the isolates of unidentified aerobic actinomycetes samples in a three-year period [19]. Unfortunately, this study did not present the clinical contexts in which these samples were taken, and most examples of* Streptomyces griseus *clinical infections come from scattered case reports such as our presented case. 

## Conclusions

It is critical for spinal surgeons to consider infection by fastidious organisms, such as* Streptomyces griseus*, on the differential diagnosis of SI joint pain, especially in patients with systemic symptoms and elevated inflammatory laboratory markers. If mycelia are noted on initial aspiration microscopy, antimicrobial therapy covering a wide range of systemic fungal and aerobic/anaerobic actinomyces organisms may be warranted. Furthermore, referral for evaluation of an underlying immunodeficiency disorder can be considered in patients with unknown history and findings of infectious sacroiliitis due to organisms such as *Streptomyces griseus*, *Mycobacterium*, and *Aspergillus*.
